# Impacts of Grapevine Leafroll Disease on Fruit Yield and Grape and Wine Chemistry in a Wine Grape (*Vitis vinifera* L.) Cultivar

**DOI:** 10.1371/journal.pone.0149666

**Published:** 2016-02-26

**Authors:** Olufemi J. Alabi, L. Federico Casassa, Linga R. Gutha, Richard C. Larsen, Thomas Henick-Kling, James F. Harbertson, Rayapati A. Naidu

**Affiliations:** 1 Department of Plant Pathology, Washington State University, Irrigated Agriculture Research and Extension Center, Prosser, Washington, United States of America; 2 Viticulture and Enology Program, Washington State University, Wine Science Center, 2710 Crimson Way, Richland, Washington, United States of America; University of Minho, PORTUGAL

## Abstract

Grapevine leafroll disease (GLD) is an economically important virus disease affecting wine grapes (*Vitis vinifera* L.), but little is known about its effect on wine chemistry and sensory composition of wines. In this study, impacts of GLD on fruit yield, berry quality and wine chemistry and sensory features were investigated in a red wine grape cultivar planted in a commercial vineyard. Own-rooted Merlot vines showing GLD symptoms and tested positive for *Grapevine leafroll-associated virus 3* and adjacent non-symptomatic vines that tested negative for the virus were compared during three consecutive seasons. Number and total weight of clusters per vine were significantly less in symptomatic relative to non-symptomatic vines. In contrast to previous studies, a time-course analysis of juice from grapes harvested at different stages of berry development from symptomatic and non-symptomatic vines indicated more prominent negative impacts of GLD on total soluble solids (TSS) and berry skin anthocyanins than in juice pH and titratable acidity. Differences in TSS between grapes of symptomatic and non-symptomatic vines were more pronounced after the onset of *véraison*, with significantly lower concentrations of TSS in grapes from symptomatic vines throughout berry ripening until harvest. Wines made from grapes of GLD-affected vines had significantly lower alcohol, polymeric pigments, and anthocyanins compared to corresponding wines from grapes of non-symptomatic vines. Sensory descriptive analysis of 2010 wines indicated significant differences in color, aroma and astringency between wines made from grapes harvested from GLD-affected and unaffected vines. The impacts of GLD on yield and fruit and wine quality traits were variable between the seasons, with greater impacts observed during a cooler season, suggesting the influence of host plant × environment interactions on overall impacts of the disease.

## Introduction

Viruses produce a wide range of symptoms in susceptible plants, modulate host metabolic pathways and cause significant losses to crop yield and quality of produce [1–4]. The extent of negative impacts of viruses, however, depends on specific virus-host combinations, virulence of the virus, cultivar susceptibility and plant age at which infection has occurred. Currently, a great deal of knowledge on compatible plant–virus interactions and impacts of virus infections on plant life-history traits are available from annual plants [5]. In contrast, studies on the consequences of virus infections in perennial plants are very limited. Unlike annual plants, perennial plants live for many years and viruses persist throughout the lifespan of these plants. Consequently, the dynamics of compatible host-virus interactions in perennial plants may be more complex and subject to an array of environmental variables and developmental cues across consecutive seasons compared to annual plants [6].

Grapevine (*Vitis* spp.) is a clonally propagated, perennial fruit crop that is cultivated worldwide [7]. In addition to yield, fruit quality is an important trait in grape production due to multiple uses of grapes for making juice, jams, jellies and wine. The grape berry is a non-climacteric fruit and its development proceeds in two successive growth stages consisting of berry formation and ripening separated by a lag phase commonly referred to as *véraison* [8]. Grape ripening is a complex process and berry quality traits are responsive to environmental cues and viticultural practices [9]. In addition, grapevine exhibits the highest seasonal variation in yield compared to other crops and this variation across seasons was suggested to be a response to Genotype (G)-by-Environment (E) interactions [10]. Thus, influences of G × E interactions need to be taken into account for a better understanding of the complex interplay between grapevine-virus interactions under the vagaries of field conditions.

Grapevines are susceptible to a wide range of virus and virus-like agents [11]. Among them, grapevine leafroll disease (GLD) is considered a serious threat to wine grapes (*V*. *vinifera*) across many grapevine-growing regions [12]. GLD is a complex viral disease producing contrasting symptoms in red- and white-berried cultivars [13]. Several genetically distinct closteroviruses, designated as grapevine leafroll-associated viruses (GLRaVs, family *Closteroviridae*), were documented in grapevines worldwide [11], [14]. Although GLRaVs have been implicated in GLD symptoms, strains of GLRaV-2 and GLRaV-7 can cause asymptomatic infections in grapevines [15], [16]. Among the currently known GLRaVs, GLRaV-3 has been reported in almost all grapevine-growing regions worldwide [17]. Likewise, GLRaV-3 was found to be more prevalent compared to GLRaV-1, -2, and -4 and its strains GLRaV-5 and -9 documented in Washington vineyards [18]. In addition to transmission via vegetative propagation materials, GLRaV-3 can be transmitted in a semi-persistent manner by mealybugs (Hemiptera: Pseudococcidae) and scale insects (Hemiptera: Coccidae) [19], [20]. One of the unique features of GLD is that symptoms become apparent on mature leaves during post-*véraison*, even though GLRaV-3 is distributed systemically and can be detected throughout the season [12].

Previous studies conducted with red-berried *V*. *vinifera* cultivars in several grapevine-growing regions have shown that GLD causes significant losses in both yield and quality of the fruit [21–26]. These studies were primarily conducted with grafted vines under varying environmental conditions and viticultural practices, with the data largely obtained from a single season at, or close to, typical commercial harvest date regimes. Although these studies provided overall end-point impacts of GLD in a single season, the heterogeneity of field conditions under which the experimental data was collected makes it difficult to dissect rootstock-conferred influences from virus disease impacts. Conversely, own-rooted vines can provide an alternative to elucidate cultivar- and site-specific influences for a better understanding of compatible host-virus interactions. Previous studies have not addressed grape compositional changes in virus-infected grapevines during berry development and ripening processes [21–26]. Consequently, the spatio-temporal dynamics of grape quality parameters across berry developmental stages during asymptomatic pre-*véraison* and symptomatic post-*véraison* stages of GLD should be examined to discern whether disease impacts occur throughout berry development and ripening processes or become apparent concomitant with symptoms in a developmental stage-specific manner. Moreover, detailed experimental evidence is lacking so far on the effects of GLD on wine chemistry and sensory attributes of wines. Since grape ripening and berry quality are complex traits subjected to environmental cues and viticultural practices and variable across seasons, the influence of G × E interactions need to be considered for a comprehensive understanding of negative impacts of virus diseases on fruit yield and quality of grapes and wines. Using a set of grapevines in the same vineyard during consecutive seasons would help in examining the significance of environmental factors on impacts of GLD on fruit yield and quality of grapes and wines and gain better understanding of compatible host-virus interactions in a long-living perennial fruit crop, such a grapevine.

In this study, therefore, the impacts of GLD on fruit yield, grape quality characteristics during berry development and wine quality attributes were investigated in an own-rooted Merlot wine grape cultivar grown under commercial vineyard conditions. The results from three consecutive seasons provided a comprehensive picture of impacts of GLD from “grape to wine” and contributed to a better understanding of host plant × environment interactions on complex dynamics of host-virus interactions in a perennial fruit crop. An extended abstract of this study was published earlier [27]. The results of this study not only expanded our current understating of impacts of a virus disease in a perennial fruit crop, but also laid a foundation for further studies on cultivar-dependent responses to grapevine leafroll infection in distinct geo-climatic locations. This type of study would help to explore the complex responses of grapevines to viral infections and discriminate host-virus interactions from that of confounding factors in the field due to climate-related variables.

## Materials and Methods

### Ethics statement

Specific approval was obtained from the owner of a commercial vineyard for collecting the data used this study. Name of the location and owner of this private property is withheld due to confidentiality as per the grower’s request. This study did not involve endangered or protected species. All the panelists involved in sensory evaluation of wines made for this the study signed an informed consent form and the project previously approved by the WSU Institutional Review Board for human subject participation.

### Plant materials

**Fig 1:**
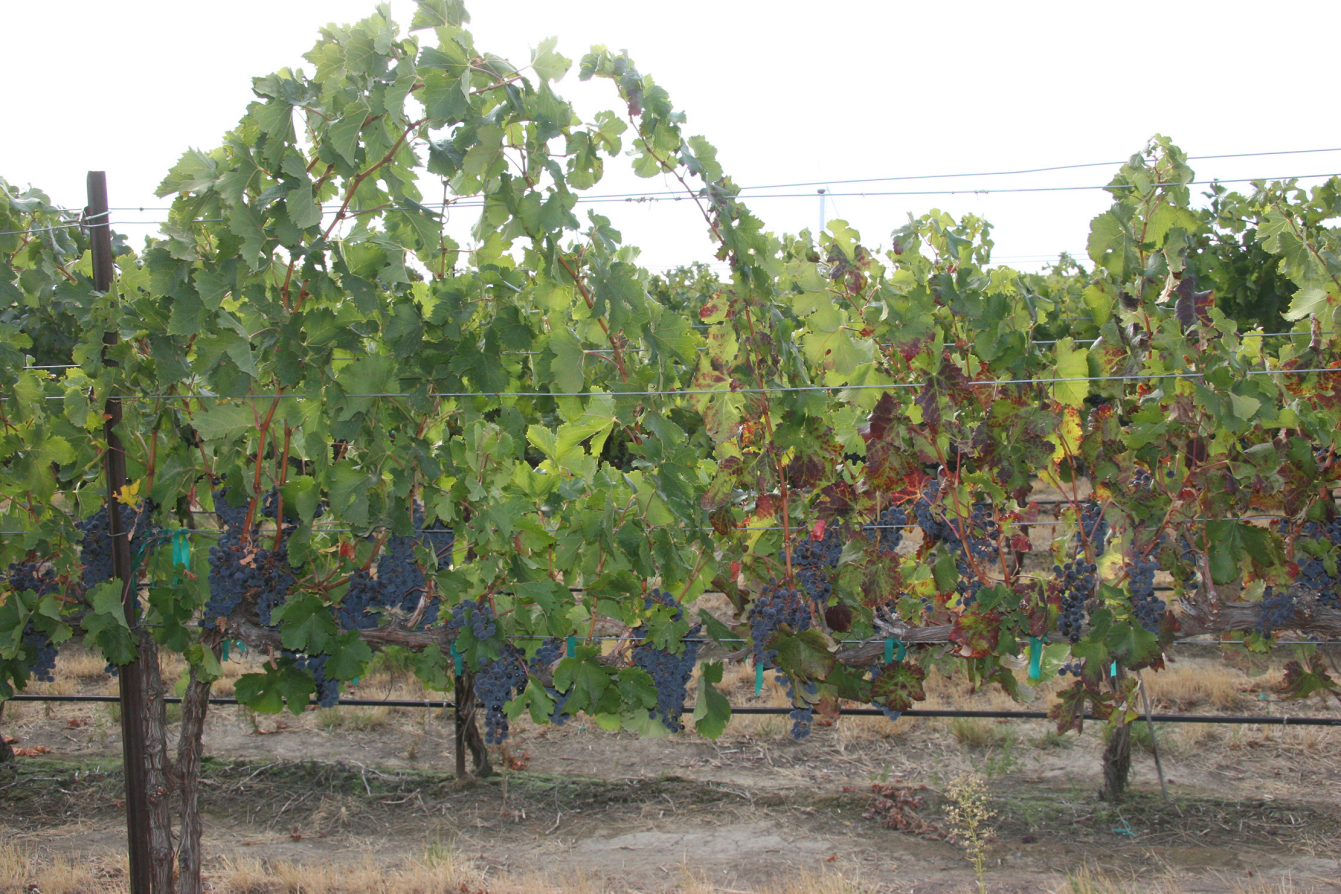
Symptoms of grapevine leafroll disease (GLD) in cv. Merlot. Merlot vines with (right) and without (left) GLD symptoms. Symptomatic vines were positive for GLRaV-3 and showed less vigorous growth and lower fruit yield compared to non-symptomatic vines.

The study was carried out during the 2009, 2010 and 2011 growing seasons in a commercial vineyard block planted in 1998 with own-rooted wine grape cv. Merlot. The vineyard block was located near Prosser, in the Yakima Valley AVA Region II of eastern Washington State (46.2°N latitude, 119.8°W longitude) and maintained by the grower using standard viticultural practices. Anecdotal evidence suggested that GLD was introduced into the vineyard block via planting of virus-infected cuttings. For this study, symptomatic and adjacent non-symptomatic vines were selected within the vineyard block by excluding vines at the perimeter to avoid possible ‘edge-effect’ on the experimental data. Vines showing typical symptoms of GLD and those without GLD symptoms (Fig 1) were selected in different rows for this study. Previously, high-throughput sequence analysis of small RNAs from the same vineyard block revealed the presence of GLRaV-3 only in symptomatic Merlot vines and three viroids (*Hop stunt viroid*, *Grapevine yellow speckle viroid 1* and *Grapevine yellow speckle viroid 2*) in both symptomatic and non-symptomatic vines [28]. Thus, vines used in this study were tested by RT-PCR as described earlier to ensure that symptomatic vines are positive for GLRaV-3 and non-symptomatic vines are negative for the virus [28].

### Fruit yield

Twelve pairs of vines, each pair consisting of one symptomatic and adjacent non-symptomatic vine in the same row (Fig 1), were selected for collecting data on fruit yield. Grapevines were selected such that each pair of vines is located in different rows in the vineyard block. Grapes were harvested from individual vines at commercial harvest on 2^nd^ October, 4^th^ October and 21^st^ October in 2009, 2010, and 2011, respectively. Clusters from each vine were harvested separately, counted and their cumulative weight determined using a digital SVI-50C weighing scale (Acculab, Edgewood, NY, USA).

### Weight of cane pruning

All canes from individual vines used for harvesting fruit were pruned per grower’s specifications in the winter following each growing season. The combined weight of from each vine was measured using the digital SVI-50C weighing scale.

### Berry sampling and biochemical analysis

Berries were harvested during 2009, 2010, and 2011 seasons from the same set of twelve pairs of symptomatic and non-symptomatic vines used for fruit yield measurement. Berries were collected at weekly intervals beginning from Eichhorn-Lorenz (E-L) stage 33 until commercial harvest, except during *véraison*, when berry samples were harvested at 3-day intervals. At each sampling time, berries were harvested randomly from individual clusters across the cordon from the eastern and western sides of the canopy, as well as from north to south, in order to exclude sunlight and temperature effects on the composition of berries [29] and to minimize cane-to-cane variations across the cordon. Due to berry developmental variability within and between clusters of the same vine, berries were collected for consistency between treatments from middle portion of individual clusters throughout the sampling period. The same number of berries was harvested from each vine with intact pedicels to prevent changes in berry components due to injury. At each time point, berries from symptomatic and non-symptomatic vines were harvested during the same time period between 8:00 and 10:00AM and stored in plastic bags in ice-containing coolers until transportation to the laboratory. Prior to analysis, berries from symptomatic and non-symptomatic vines were weighed individually and the weights recorded and added to the cumulative weight for each vine at harvest. Thereafter, the berries were pooled separately and subsequently divided randomly into five replicates of up to 50 berries for each category. The number of berries per replicate was kept constant between the two categories at each sampling point, but varied between sampling points depending on the size and physiological state of berries. Thus, an average of 50 berries per replicate in both categories was used during pre-*véraison* due to their small size and an average of 35 berries per replicate used during post-*véraison* due to their large size. The same sampling pattern was maintained for all three seasons.

Each berry was gently destalked and fresh weight of berries in each replicate was measured using a digital VI-350 weighing scale (Acculab, Edgewood, NY, USA) and homogenized in an A11 analytical grinding mill (IKA^®^ Works, Inc., Wilmington, NC, USA). Following centrifugation (5,000 x *g* for 6 min, 5°C), the clear supernatant of the homogenate was used for further analysis. Total soluble solids (TSS) were measured using a PAL-1 digital pocket refractometer (Atago Co., Tokyo, Japan) and expressed as °Brix (percent of soluble sugars). Titratable acidity (TA) was determined by direct titration with 0.1 N NaOH to an endpoint of pH 8.2 using a DL50 Rondolino Autotitrator (Mettler Toledo Inc., Columbus, OH, USA) and expressed as g/L of tartaric acid. The juice pH was measured with a MP225 pH meter (Mettler Toledo Inc., Columbus, OH, USA) and total anthocyanins were measured according to the protocol developed by Iland et al. [30].

### Winemaking

Non-symptomatic grapevines were used as the standard for ripeness determination and harvest occurred when the fruit juice of grapes from non-symptomatic vines reached ~24°Brix. Grape clusters were harvested manually from symptomatic and non-symptomatic vines on 2^nd^ October, 4^th^ October, and 21^st^ October of 2009, 2010, and 2011, respectively. Grapes were harvested separately from symptomatic and non-symptomatic vines on the same day. The harvested fruit was transported to the Research Winery at the Washington State University—Irrigated Agriculture Research and Extension Center (WSU-IAREC), Prosser, WA. Fruit from each category was pooled and divided into three replicates, with each replicate containing approximately 70 kg (~150 lbs) of total fruit.

Grape clusters from each replicate were crushed and destemmed separately using a Mearelli crusher/destemmer (Cinquemiglia, Città di Castello, Italy). Potassium metabisulfate (SO_2_) was added at the rate of 50 ppm immediately after crushing. Six hours after crushing, the musts were inoculated with 30 g/hL of commercial dry yeast (Lalvin EC-1118, Lallemand Inc., Montreal, Canada). Cap management consisted of two daily manual punch-downs on the fermenting musts until pressing. Total skin contact time was 10 days. Sugar consumption and temperature were monitored daily with a hand-held densitometer calibrated for Brix (DMA 35N, Anton Paar, Graz, Austria) and residual sugars were measured at the later stages of fermentation by the Rebelein method [30]. Ten days after the completion of alcoholic fermentation (reducing sugars < 2 g/L), 1 g/hL of *Oenococcus oeni* bacterium strain Lalvin VP-41 was added to the wines to undergo malolactic fermentation (MLF). The monitoring of MLF and reduction of malic acid was performed as described previously [31]. About 35 days post-crush, or after the completion of MLF (malic acid concentration s < 0.1 g/L/), the wines were racked off the lees, cold-stabilized (90 days 2 ± 2°C) and adjusted for free SO_2_ to 30 ppm. Following adjustment of SO_2_ to final target molecular concentration of 0.5 ppm, the wines were bottled in 750 mL bottles under screw-cap leaving a 16 mL headspace with the aid of a capping machine (Technovin TVLV, Saxon, Switzerland) and stored horizontally at 10 ± 2°C until needed.

### Wine analysis

Anthocyanins, small and large polymeric pigment and tannin contents of the wines were measured as described previously [32]. Alcohol content was measured with a NIR-based method using an Alcolyzer Wine M/ME Wine Analysis System (Anton Parr, Graz, Austria).

### Sensory evaluation

Wines from the 2010 and 2011 seasons were analyzed by descriptive analysis and overall difference test, respectively. A descriptive sensory analysis was conducted on the wines of the 2010 season over the course of three weeks during June 2011. A trained panel (n = 15; 9 males and 6 females) recruited from the Prosser community, including members of staff of WSU-IAREC and two commercial wineries located in the Yakima Valley area of WA, was convened. Demographic aspects were recorded at the beginning of the first session and no information about the nature of the study was provided to the panelists in order to reduce bias. Panelists were screened for bitterness sensitivity (sensitivity to 6-n-propylthiouracil (PROP), also known as PROP status) and color blindness [33] and subsequently trained during seven consecutive sessions each lasting between 45 min to 1 hr. Terminology development occurred by consensus of the whole panel. After reviewing the standards, panelists evaluated a series of 10 commercial wines, and discussed the intensity of the different attributes relative to the standards using a 15 cm scale. Reference standards for aroma and color were used for this purpose as described earlier [31]. The overall performance of the panel and individual panelists was evaluated leading to the elimination of three panelists based on preliminary analysis of the data, interaction plots and analysis of outliers. The remaining 12 panelists (df = 11) evaluated the two experimental wines from infected and control vines. To avoid bias due to color, tulip-shaped cobalt black glasses (Libbey, Toledo, OH, USA) were used for aroma and astringency evaluation and clear wine glasses (ISO 3591:1977) used for color evaluations. Panelists assessed the wines in individual booths under white light, at the Sensory Laboratory of WSU-IAREC, Prosser. Each panelist was provided with deionized filtered water (Easy Pure II, Thermo Scientific, Dubuque, IA, USA) and unsalted crackers (Great Value, Bentonville, AR, USA) for palate cleansing in between evaluations. Twenty-five mL aliquots of wine at room temperature (20 ± 1°C) were poured into wineglasses coded with three-digit random numbers and covered to trap volatiles. Wines were presented using a complete randomized design including the three replicates for each wine during four evaluation sessions. Results were collected on ballots and manually decoded with a ruler. All panellists involved in the study signed an informed consent form and the project previously approved by the WSU Institutional Review Board for human subject participation.

A forced-choice triangle test was selected to explore potential differences among the wine produced from symptomatic and non-symptomatic vines during the 2011 season. The overall difference test was selected for the 2011 wines due to initial assessment of the wines showing relatively minor differences between treatments. Previous informal pre-screening of the wines carried out by three experienced wine tasters revealed that wines from the two treatments, and their replicates (2 × 3) were free of off-odors or other taint aromas and thus were suitable for sensory evaluation. Since the main goal of the triangle test is to determine an overall sensory difference between the wines, special emphasis was placed on the control of the type I error (i.e. α-risk). All panelists were recruited from the WSU-IAREC community. Demographic aspects such as age, sex, and red wine frequency consumption were recorded at the beginning of the test. Thirty-three consumers (n = 33; 17 males and 16 females) aged between 21 and 60 years participated in the test. Further demographic information indicated that members of the consumer panel were composed by light to moderate wine consumers with about 79% of them declaring that they drink wine at least once a month and 42% drinking red wine at least once a week (data not shown). Panelists were briefly introduced to the mechanics of the triangle test but no information about the nature of the study was provided in order to minimize bias. Panelists were tested for visual disorders as described above and results of this test indicated that none of the panelists had color deficiencies. Aliquots of 25-mL coded wines, consisting on two treatments (symptomatic and non-symptomatic) and three replicates per treatment, were presented in a complete randomized design at room temperature (20 ± 1°C) as described for the 2010 wine evaluations and panelists were presented with three samples per flight, for a total of two flights. In the first flight, the wines were presented in transparent glasses. In the second flight, a new set of three wines was presented in black glasses. Evaluations were recorded on a ballot designed according to Meilgaard et al. [34]. As in the case of the descriptive analysis, all panelists involved in the study signed an informed consent form previously approved by the Washington State University Institutional Review Board for human subject participation.

### Data treatment and statistical analysis

Data for each parameter were obtained in replicates per treatment and subjected to two-way analysis of variance (ANOVA) in order to assess the influence of each treatment (infected and uninfected) on the parameters being evaluated. We also checked for season effects as well as ‘treatment × season’ interaction effects. These analyses were carried out using the SigmaPlot statistical software for Windows, version 11.0 (Systat Software, Inc., Germany). The confidence levels of all analyses were set at 95% and values with *p* ≤ 0.05 were considered significant. Statistically significant treatment means were then separated using appropriate tests. The sensory-trained panel data for the 2010 wines was analyzed by a three-way mixed-effect analysis of variance (ANOVA) with replications. Panelists were considered as random effect and treatments (symptomatic and non-symptomatic) and wine replicates and their interactions were treated as fixed effects. The analysis was carried out using XLSTAT v. 2011 (Addinsoft, Paris, France). A 5% level for rejection of the null hypothesis was used for each experiment and Tukey’s Honestly Significant Difference (HSD) test was used as a *post-hoc* comparison of means. The whole data set with replicates was also analyzed by a principal component analysis and confidence ellipses (95% certainty), calculated using the multivariate Hottelling test, were constructed using the software R Version 2.1.1 (Foundation for Statistical Computing, Vienna, Austria). Only components with Eigen-values > 1 were retained. For the forced-choice triangle test performed on the 2011 wines, the statistical power (1-ß) and ß values were obtained using the Test Sensitivity Analyzer [34]. Assigning a α-risk of 0.05, a total of at least 16 correct responses were needed for a proportion of distinguishers of 30% and a ß-risk of 0.24. In other words, 16 or more correct responses were needed to prove a difference between the two treatments at α-level of 0.05. The statistical power (1-ß) for this test was 0.76 [34]. Storage and graphic generation of the data set were then achieved using XLStat (Addinsoft, Paris, France).

## Results

### General meteorology

Meteorological data was retrieved from the Roza weather station (46.3°N latitude, 119.7°W longitude) of the Washington Agricultural Weather Network (AgWeatherNet; http://weather.wsu.edu/awn.php), located close to the commercial vineyard block (46.2°N latitude, 119.8°W longitude). The meteorological data indicated that cumulative growing degree-days were higher during the 2009 season compared to the 2010 and 2011 seasons (S1 Fig). Based on this data, 2009 was considered a warmer season compared to relatively cooler conditions that prevailed during the 2010 and 2011 seasons.

### Effect of GLD on fruit yield and vine vigor

Previous studies indicated that Merlot vines showing GLD symptoms were positive for GLRaV-3 and non-symptomatic vines negative for the virus [18], [28]. In the present study, grapevines with and without GLD symptoms (Fig 1) were retested by RT-PCR during each season to ensure that vines with symptoms are positive for GLRaV-3 and those without symptoms are negative for the virus. In the 2009 and 2010 seasons, twelve vines with GLD symptoms and tested positive for GLRaV-3 and equal number of non-symptomatic vines adjacent to symptomatic vines and tested negative for the virus were selected for collecting data on fruit yield and weight of cane pruning. In the 2011 season, only eight pairs were used since four non-symptomatic vines were tested positive for GLRaV-3 indicating temporal disease spread.

The number and weight (kg) of berry clusters per vine were compared at commercial harvest during the 2009, 2010, and 2011 seasons (Table 1). The results showed reduction in number and total weight of clusters and weight of cane pruning per vine in symptomatic vines relative to non-symptomatic vines in all three seasons (Table 1). Average cluster weight per vine was reduced between 16 to 28% in symptomatic vines during all three seasons. Similarly, the number of clusters per vine showed reduction between 14 to 20% in symptomatic vines during all three seasons (Table 1). Cane pruning weight per vine measured during the 2010 and 2011 seasons was reduced by 11.2% and 24.3%, respectively, compared to pruning weight from non-symptomatic vines (Table 1). Among the three parameters studied, only the number of clusters per vine showed significant treatment and seasonal effects (Table 1), as determined by a two-way ANOVA using the Holm-Sidak test [35]. Based on these results, it can be concluded that GLD affects vine vigor and fruit yield in own-rooted cultivar Merlot under the arid climate conditions of eastern Washington State.

**Table 1:** Impacts of grapevine leafroll disease (GLD) on vine vigor and fruit yield and quality. Multi-season effect of GLD on yield parameters, vine vigor and basic fruit composition of own-rooted cv. Merlot vines at commercial harvest.

Variable	Treatment means by season^γ^					
	2009		2010		2011	
	Non-symptomatic	Symptomatic	Non-symptomatic	Symptomatic	Non-symptomatic	Symptomatic
Yield (kg/vine)^α^	4.70	3.39	4.19	3.52	5.68	4.51
Bunch/vine (n) ^α^	90*^,a^	76*^,b^	86*^,a^	70*^,b^	116*^,a^	99*^,b^
Pruning weight (g/vine) ^α^	NA	NA	315.0	279.6	359.3	272.0
TSS (Brix)^β^	24.8*^,a^	23.3*^,b^	25.0*^,a^	23.1*^,b^	23.5*^,a^	22.5*^,b^
Titratable acidity (g/L) ^β^	5.47*^,b^	6.10*^,a^	6.40*^,b^	6.76*^,a^	4.35*^,b^	4.69*^,a^
pH ^β^	3.65*^,a^	3.58*^,b^	3.34*^,a^	3.33*^,b^	3.65*^,a^	3.55*^,b^

^α^Data represents means of raw data from 12 pairs of non-symptomatic (uninfected) and symptomatic (GLD-affected) vines for 2009 and 2010 seasons and eight pairs of vines for the 2011 season due to new infections of four non-symptomatic vines as determined by RT-PCR.

^β^Data represents means of raw data from fruit triplicates taken from fruit lots from non-symptomatic and symptomatic vines at commercial harvest.

^γ^Means followed by an asterisk (*) differ statistically (*p* ≤ 0.05) and alphabetical letters were used to separate means for each significant treatment effect comparison. Significant season effects (*p* ≤ 0.05) were obtained for all variables except yield and pruning wood weight but no significant ‘Treatment × Season’ effects were found in all cases. NA, data not taken.

### Effect of GLD on fruit composition

Analysis of juice from grapes harvested at different intervals revealed consistent and significant (*p* < 0.05) reduction in TSS of grapes from symptomatic vines relative to non-symptomatic vines in all three seasons (Table 1). The reduction in TSS ranged between 4% and 8%, with greater reduction observed during the 2010 season (7.7%), followed by the 2009 season (6.2%) and the least reduction observed during the 2011 season (4.1%). In contrast, juice pH was lower in grapes from symptomatic vines relative to grape samples from non-symptomatic vines in the 2009 (-1.9%) and 2011 (-2.8%) seasons than in the 2010 season (Table 1). With regard to TA, higher values were obtained in all three seasons with juice of grapes obtained from symptomatic vines relative to non-symptomatic vines. However, the percent change in TA value was higher in the 2009 season (11.5%), followed by the 2011 (7.8%) and 2010 (5.6%) seasons (Table 1). As expected, an inverse relationship between pH and titratable acidity was observed in fruit juice from symptomatic relative to non-symptomatic vines during all three seasons. The data on TSS, pH and TA showed significant season effects but no ‘treatment × season’ interaction effects were observed as determined by two-way ANOVA (Table 1).

### Impact of GLD on fruit composition during berry development

**Fig 2:**
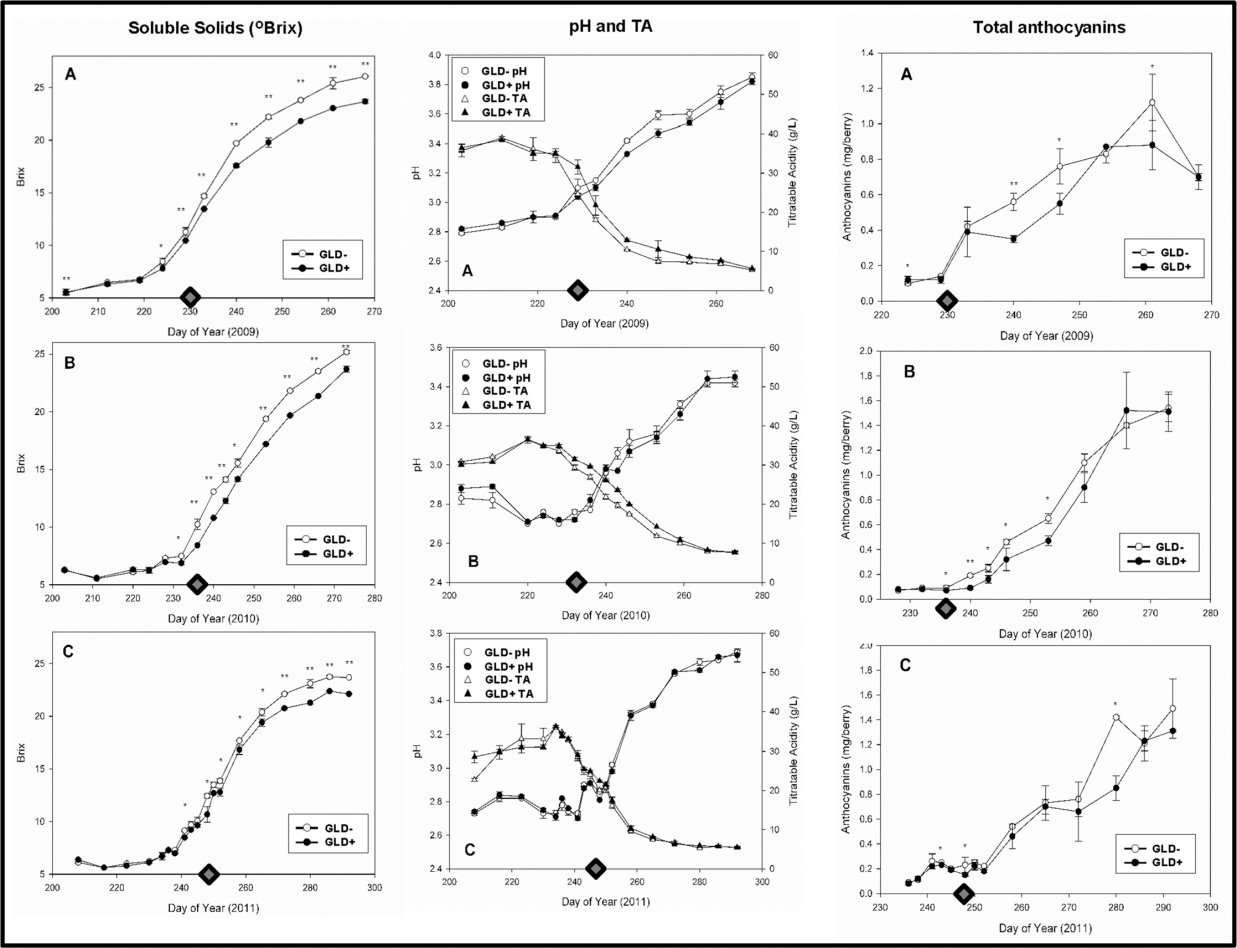
Impacts of grapevine leafroll disease (GLD) on fruit quality. Time-course analyses of impacts of GLD on fruit maturity indices (total soluble solids, pH, titratable acidity and total anthocyanins) in own-rooted wine grape cv. Merlot during (A) 2009, (B) 2010 and (C) 2011 seasons. Line drawings with open circles represent samples from non-symptomatic (healthy) vines while line drawings with colored circles represent samples from symptomatic (infected) vines. Each data point represents means of five replicates per treatment. Approximate date of *véraison* in each season is denoted by the grey diamond. Statistical significance was determined by one-way ANOVA: (* = *p* ≤ 0.05; ** = *p* ≤ 0.001).

GLD symptoms begin to appear soon after *véraison* even though the virus can be detected in vines throughout the season (Naidu et al., unpublished results). Therefore, experiments were conducted to study whether impacts of the disease on fruit chemistry can occur only after *véraison* or throughout berry developmental and ripening processes. For this purpose, grapes were sampled at defined stages of berry development and ripening and TSS, pH, TA and anthocyanins compared between symptomatic and non-symptomatic vines. The results (Fig 2) indicated that statistically significant (*p* < 0.05) differences in TSS, TA and pH were observed between berry samples from symptomatic and non-symptomatic vines during post-*véraison*, but not during pre-*véraison*, and these differences became more pronounced throughout berry ripening until harvest (Fig 2). Similarly, statistically significant differences (*p* < 0.05) in anthocyanins of berries from symptomatic and non-symptomatic vines were observed during the linear phase of berry ripening during post-*véraison* (Fig 2). However, differences in anthocyanins between berries of symptomatic and non-symptomatic vines were less pronounced at the time of commercial harvest (Fig 2).

A comparison of impacts of GLD on fruit quality attributes across all three seasons revealed that negative effects of the disease were more apparent during post-*véraison* than pre-*véraison* (Fig 2). Although the pattern of GLD impacts was similar in all three seasons for each of the quality attributes measured, the magnitude of impacts on each of the quality parameters studied was variable between seasons, with higher negative impacts of GLD on fruit composition occurring during cooler seasons.

### Effect of GLD on basic wine and phenolic composition

Wines produced from symptomatic and non-symptomatic vines were compared for basic wine attributes, including acidity (pH and TA), alcohol content, anthocyanins, small polymeric pigments (SPP), large polymeric pigments (LPP), iron-reactive phenolics, and tannins. These parameters were analyzed at the time of bottling as described below.

### Wine titratable acidity and alcohol

Both TA (g/L) and alcohol content (% ethanol v/v) showed significant treatment and season effects for all three seasons (Table 2). In contrast, pH showed significant season effect but the treatment effect was only significant for the 2010 wines (Table 2). Unlike juice pH of the fruit (Table 1), contrasting patterns were obtained for wine pH, in that average pH ranges were higher for wines produced from non-symptomatic vines in the 2010 and 2011 seasons, whereas the opposite was observed for the wine of the 2009 season (Table 2). The same was the case for TA, which was greater for wines produced from symptomatic vines during the 2009 and 2011 seasons, but was found in greater concentration in wines produced from non-symptomatic vines in 2010 season (Table 2). Analysis of season effects showed contrasting patterns of seasonal variations for pH and TA. Whereas average pH ranges were, in descending order of magnitude, highest in wines produced during the 2009 season followed by the 2011 and 2010 seasons, TA concentrations were highest in 2010, followed by 2011 and 2009 seasons (Table 2). Percent ethanol concentration (v/v) of finished wines showed a downward trend (2009 > 2010 > 2011) during the three seasons. However, whereas 2009 wines were significantly different from 2010 and 2011 wines, ethanol concentrations of 2010 and 2011 wines were not statistically different (*p* ≤ 0.05) from each other (Table 2). These results demonstrate that, whereas GLD showed consistent negative impacts on alcohol content of wines made with grapes from symptomatic vines relative to non-symptomatic vines regardless of the season, its effect on wine acidity were dependent on the season (Table 2; S1 Fig).

**Table 2:** Impact of grapevine leafroll disease (GLD) on wine composition. Multi-season effect of GLD on wine composition of own-rooted cv. Merlot vines.

Variable^α^	Treatment means by season^γ^					
	2009		2010		2011	
	Non-symptomatic	Symptomatic	Non-symptomatic	Symptomatic	Non-symptomatic	Symptomatic
Alcohol (% v/v)	14.98*^,a^	13.80*^,b^	14.80*^,a^	12.86*^,b^	14.01*^,a^	13.24*^,b^
Titratable acidity (g/L)	3.30*^,b^	3.83*^,a^	6.21*^,a^	5.95*^,b^	5.03*^,b^	5.30*^,a^
pH	3.84	3.91	3.42*^,a^	3.38*^,b^	3.59	3.56
Total anthocyanins (mg/L)	314*^,a^	243*^,b^	670*^,a^	602*^,b^	538*^,a^	507*^,b^
Small polymeric pigments (A_520_)	0.79*^,b^	0.92*^,a^	0.95	0.92	1.04	1.09
Large polymeric pigments (A_520_)	0.84*^,b^	1.15*^,a^	1.29*^,a^	0.87*^,b^	0.61	0.61
Tannins (mg/L)	446	509	1108*^,a^	805*^,b^	520	559

^α^Data represents means of raw data from small-lot wine triplicates produced from non-symptomatic (healthy) and symptomatic (GLD-affected) fruits lots.

^γ^Means followed by an asterisk (*) differ statistically (*p* ≤ 0.05) and alphabetical letters were used to separate means for each significant treatment effect comparison. Significant season effects (*p* ≤ 0.05) were obtained for all variables but no significant ‘Treatment × Season’ effects were found in all cases.

### Wine phenolics

Analysis of wine phenolics showed significantly higher concentrations of anthocyanins in wines produced from non-symptomatic vines relative to symptomatic vines in all three seasons. However, whereas concentrations of tannins were significantly higher in wines from grapes of non-symptomatic vines in 2010, they were slightly lower in wines from the same set of vines during the 2009 and 2011 seasons (Table 2). A similar trend was found for SPP (A_520_), but a significant treatment effect was observed only for the 2009 wines for this variable. Large polymeric pigments, LPP (A_520_) were significantly lower for the 2009 wines from grapes of non-symptomatic vines, higher for the same set of wines in the 2010 season and equal for both sets of wines for the 2011 season (Table 2). An assessment of the season effects showed that in general the 2010 wines had higher anthocyanin, LPP and tannin content relative to wines from the other two seasons (Table 2). These results showed that among the wine phenolics parameters measured in this study, negative impacts of GLD were more apparent and consistent for wine anthocyanin regardless of the season whereas GLD effect on tannins, SPP and LPP were largely season-dependent. The results also suggest that even though the time-course progression of negative impacts of GLD on berry skin anthocyanins appears to dissipate at the time of harvest (Fig 2), differences observed during and post-*véraison* were stably carried over to the finished wine products (Table 2).

### Sensory evaluation

**Fig 3:**
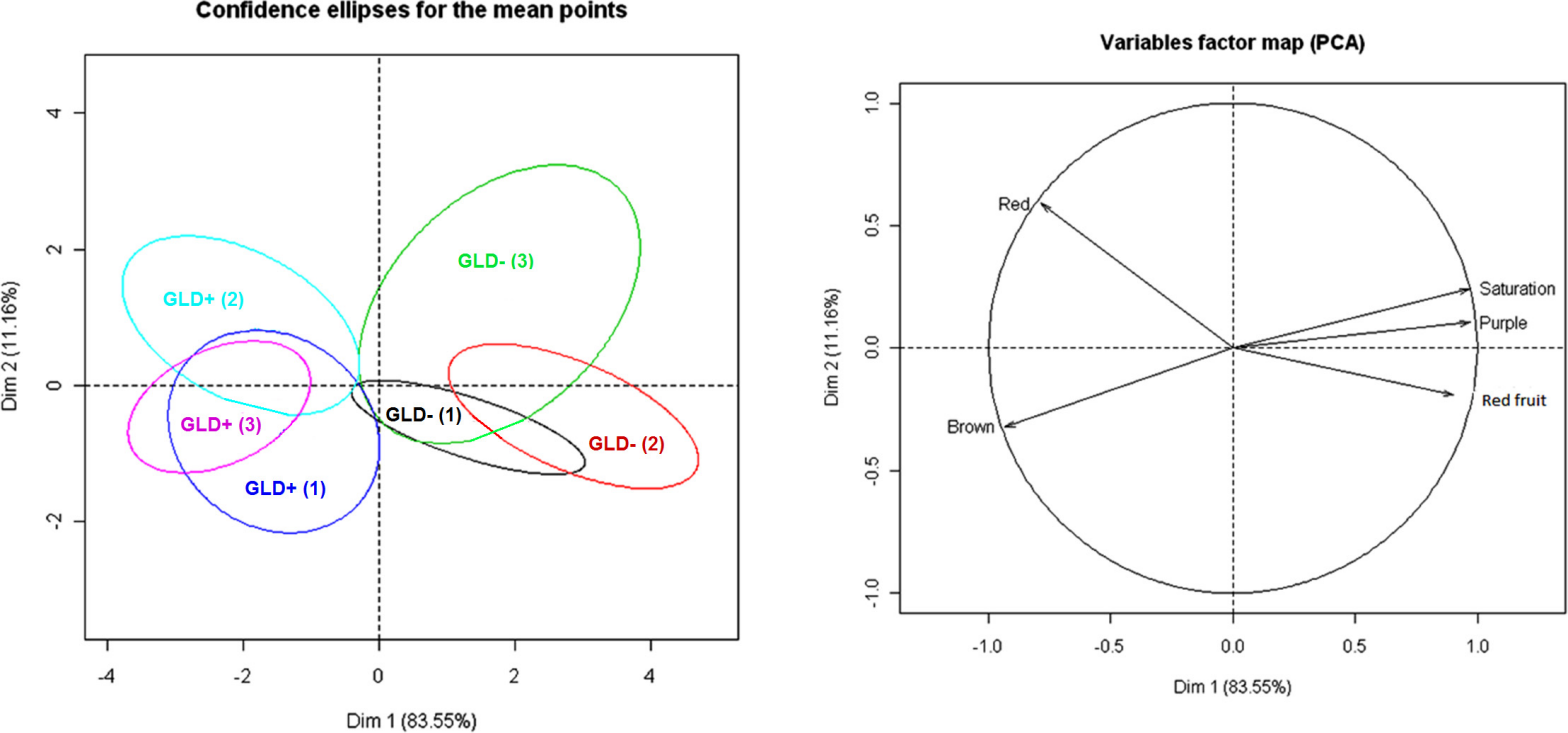
Sensory analysis of wine. Principal component analysis (PCA) plots displaying confidence intervals for each replicate of wines produced from grapes harvested from symptomatic (infected) and non-symptomatic (healthy) vines at 95% confidence according to multivariate Hottelling test.

A descriptive analysis of the 2010 wines showed significant (*p* ≤ 0.05) differences in color, aroma and astringency between wines produced from symptomatic and non-symptomatic vines (Table 3). Wines from non-symptomatic vines were perceived as being more purple in color, with less brown, and with a more saturated color, together with a higher predominance of red fruit aroma component and a lower predominance of the earthy aroma character (Table 3). The wines from non-symptomatic vines were also deemed more astringent than wines from symptomatic vines (Table 3). Since sensory data are typically non-parametric [36], principal component analysis, a multivariate technique, was carried out to examine the interrelationships between the different variables and to allow the spatial separation of the wines from symptomatic and non-symptomatic vines (Fig 3A). Wines produced from non-symptomatic vines were defined by the descriptors saturation, purple and red fruit aroma, whereas wines from symptomatic vines were defined by red and brown component (Fig 3B). Astringency and earthy aromas were not included due to the fact that a one-way ANOVA found no statistical differences between the two treatments for these two attributes (data not shown). Confidence ellipses showed minimal overlap occurring only between a pair of wine replicate from non-symptomatic and symptomatic vines (Fig 3A). The lack of overlapping ellipses for wines from both treatments indicates that, in the sensory space constructed with the two principal components, wines from symptomatic and non-symptomatic vines were different from a sensory perspective, with a confidence of 95% as calculated by the multivariate Hotelling test.

**Table 3:** Effect of grapevine leafroll disease (GLD) on wine sensory attributes of Merlot vines. A three-way mixed effect analysis of variance and mean separations along a 15-cm line scale was performed. Analyses were based on evaluations made by sensory trained panelists (n = 12) and data obtained for each variable represents mean values of raw data from wine triplicates from control and infected vines evaluated during the 2010 season.

Sensory variables	Non-symptomatic ^γ^	Symptomatic
Purple color	9.47*^,a^	6.81*^,b^
Red color	7.36*^,b^	9.01*^,a^
Overall color saturation	10.97*^,a^	7.75*^,b^
Red fruit aroma	7.01*^,a^	5.01*^,b^
Earthy aroma	4.40*^,b^	5.85*^,a^
Astringency	12.11*^,a^	10.31*^,b^

^γ^Means followed by an asterisk (*) differ statistically (*p* ≤ 0.05) and alphabetical letters were used to separate means for each significant treatment effect comparison. Significant panelist and ‘Panelist x Treatment’ effects (*p* ≤ 0.05) were also obtained for all variables.

**Fig 4:**
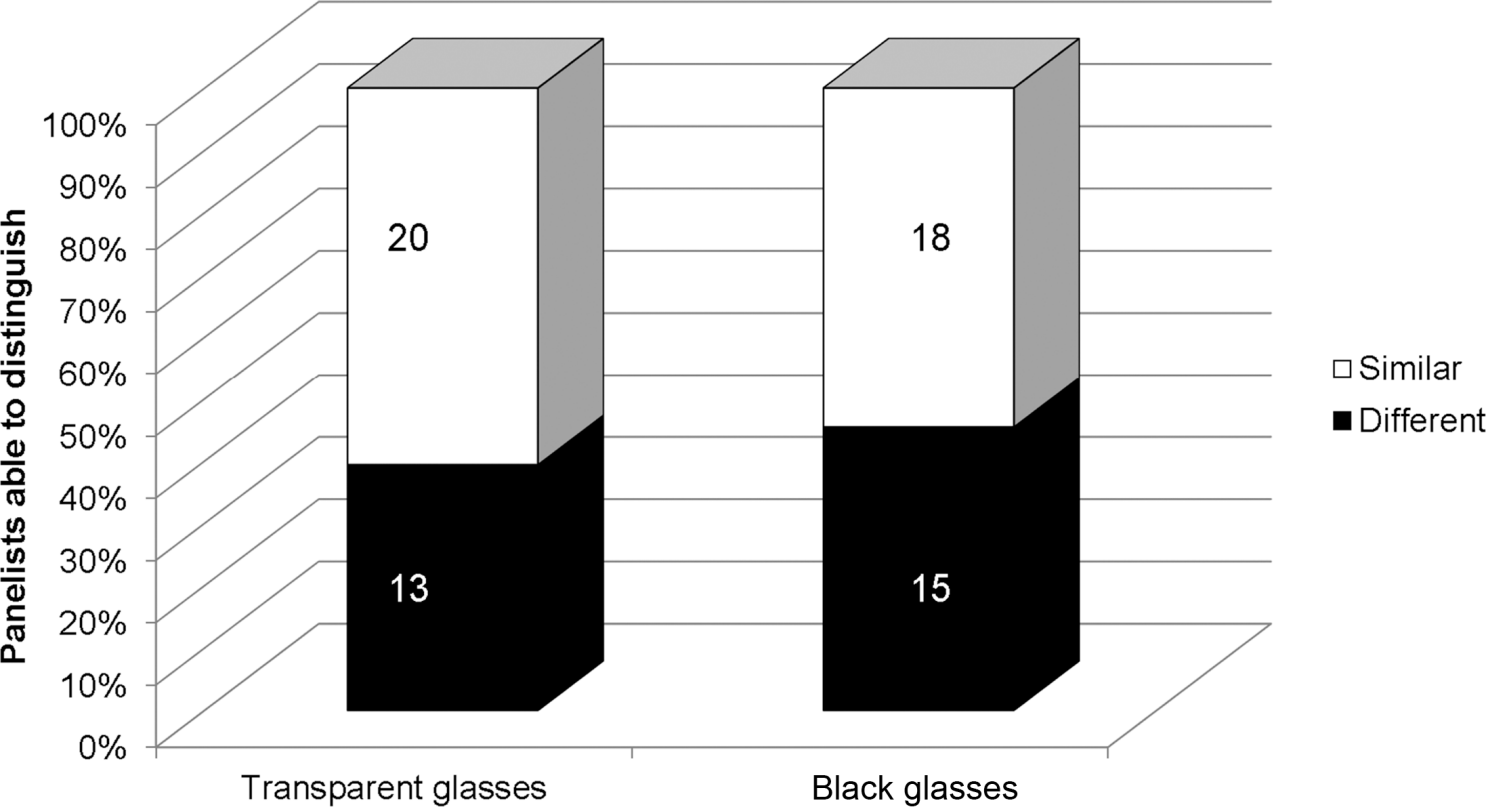
Sensory evaluation of wine. Forced-choice triangular test conducted on triplicate wines produced from non- symptomatic (healthy) and symptomatic (infected) own-rooted cv. Merlot vines. Thirty-three panelists (17 males and 16 females) evaluated wines presented in transparent and black tulip-shaped glasses for overall differences in color, aroma and astringency attributes. Sixteen panelists are required to correctly distinguish between the wine treatments in order to achieve statistical significance (*p* ≤ 0.05) [34]. Actual numbers of panelists able or unable to distinguish between wines from non-symptomatic and symptomatic vines are indicated on each bar.

The results of the forced-choice triangle test for the 2011 wines are shown in Fig 4. For a consumer panel of 33 individuals and assigning a α-risk of 0.05, a total of at least 16 correct responses were needed for a proportion of distinguishers of 30% and a ß-risk of 0.24. In other words, 16 or more correct responses were needed to prove a difference at α-level of 0.05. The statistical power (1-ß) for this test was 0.76 (Test Sensitivity Analyzer in: [34]). Evaluation of the 2011 wines failed the 16 correct response threshold as only 39% and 45% of the consumers were able to distinguish between wines from symptomatic and non-symptomatic vines in transparent and black glasses, respectively (Fig 4). Thus, it can be concluded that the consumer panel was unable to detect an overall difference between wines produced from symptomatic and non-symptomatic vines in the 2011 season. Taken together, a comparative analysis of the wines produced from both symptomatic and non-symptomatic vines during 2010 and 2011 seasons indicate an overall negative impact of GLD on wine sensory properties.

## Discussion

It is well documented that rootstock genotypes have a wide range of effects on scion characteristics, such as vine phenology and grape composition and responses to different biotic and abiotic stress factors [37], [38] and references cited in [37], [38]. Likewise, impacts of GLD were reported to be variable in grafted vines of different red-berried cultivars depending on the rootstock-scion combination [39], [23–26]. In contrast, the present study was conducted with grapevines grown on their own roots to elucidate compatible host-virus interactions in the absence of rootstock-conferred influences on the scion in a perennial fruit crop. The data provide an overall view of the dynamic changes in the grape berry composition, instead of a snap shot at the time of commercial harvest, and offered valuable insights on effects of viral infection on berry quality parameters. Unlike previous reports that are largely based on data collected at one time-point from a single season, this multi-season study provided holistic analyses of vintage-specific effects of virus disease impacts from “grape to wine”.

Lower fruit yield due to reduced cluster number and weight was consistently observed in virus-infected vines compared to uninfected vines in all three seasons (Table 1). Together with the data on cane pruning, which is a measure of vine vigor during the previous season, the results herein present support the overall conclusions that GLD caused negative impacts on vine performance and overall yield components, although these parameters showed variable responses between seasons. It should be noted, however, that seasonal variance in yield and vine vigor was observed in both symptomatic and non-symptomatic vines and the observed variation could likely be due to host plant x environment interactions under different meteorological conditions. This is consistent with previous studies indicating that grapevine cultivars show diverse responses in phenology and fruit yield and quality to environmental perturbations between seasons [10], [40].

Although the influence of various viticultural practices and environmental and abiotic stress factors on berry composition is well documented [41], comparatively less is known on consequences of viral infection on grape quality parameters in relation to berry development. The results presented in this study (Fig 2) indicated that developing green berries from virus-infected vines show minimal compositional changes compared to berries from uninfected vines during pre-*véraison*. In contrast, dramatic differences were observed during post-*véraison* between berries from infected and uninfected vines, suggesting that viral infection caused more significant impacts on ripening-related processes starting from *véraison*. Data from this study also suggest that, among the quality parameters measured during various stages of berry development and ripening, total soluble solids, a hallmark of berry quality, were significantly affected during post-*véraison* in all three seasons. Since sugars accumulating in berries during the log phase of their ripening are largely transported from autotrophic leaves [42], [43], our observations support the hypothesis that reduced sugar accumulation in ripening berries occurred as a consequence of declining influx of sugars from autotrophic leaves during post-*véraison*. This is plausible due to the fact that GLRaVs are phloem-limited [44] and diminished assimilate partitioning is likely a consequence of viral-induced interference of phloem translocation [45].

During the linear phase of berry ripening from *véraison*, significantly lower concentrations of anthocyanins were observed in berries of virus-infected vines compared to berries from uninfected vines (Fig 2). Reduced anthocyanins throughout fruit maturation is likely due to decreased amount of sugars in ripening berries, since a tight positive correlation has been reported between sugars and anthocyanin concentration in berries [46]. However, anthocyanins in berries from infected and uninfected vines were almost similar at commercial harvest suggesting that accumulation of anthocyanins in berries of virus-infected vines did not correlate with sugar accumulation during later stages of grape ripening. This uncoupling between anthocyanins and sugars at grape maturity can be explained by previous observations that anthocyanin accumulation during berry ripening is a two-phased process, with tight positive correlation between accumulation of sugars and anthocyanin biosynthesis during the log phase of ripening followed by a second phase where accumulation of anthocyanins and sugars are uncoupled [46], [47]. The first phase is influenced mainly by viticultural practices and source to sink balance and the second phase is strongly affected by environmental cues and seasonal conditions.

To the best of our knowledge, there are no detailed reports on the effect of GLD on wine sensory attributes. Therefore, the present study examined an overall negative impact of GLD on sensory attributes of the wines produced from symptomatic vines during the 2010 season and confirmed using both formal descriptive analysis and overall difference tasting techniques. Furthermore, the data suggest that negative impacts of GLD on the sensory properties of the wines may be exacerbated in comparatively cooler seasons indicating vintage effects (Table 3; S1 Fig). The current study also showed significant impacts of GLD on wine chemistry, especially alcohol and anthocyanins, than other components (Table 2). These impacts were variable between the three seasons, with significant differences observed between wines of the 2010 season compared to the other seasons. A detailed sensory analysis of 2010 wines (Table 3) further confirmed severe impacts of GLD on wine sensory properties. Anthocyanins have the capacity to form covalent bonds with tannins during winemaking [48] and reduced anthocyanins therefore have significant implications for both wine astringency and color stability. Even though this study showed an impact of GLD on wine aroma, specific aromatic compounds affected were not determined. Therefore, more studies are needed to further elucidate the impacts of GLD on specific aromatic compounds using analytical approaches such as GC-MS and/or GC-O. Additionally, data generated in this study may form a baseline for decision-making by winemakers for considerations of different winemaking techniques to compensate for the negative impacts of GLD by taking into account the disease status of the vineyard block. It is noteworthy, however, that in the current study, comparisons were made between wines made from grapes produced by symptomatic and non- symptomatic vines. Since disease gradients are the norm in most commercial vineyards, it will be interesting to determine if the negative impacts observed in this study can be alleviated by blending the two types of wines or mixing fruit from GLD-affected and unaffected vines before winemaking.

In summary, this is the most comprehensive study conducted so far on impacts of GLD from grape to wine. The study demonstrated that the impact of GLD was more significant on sugar production and anthocyanins than on juice pH and TA. In addition, the negative impact on sugars was more apparent during post-*véraison* than pre-*véraison*, suggesting that sugar content in ripening berries was affected due to GLD. This supports the hypothesis that reduced sugar concentration (or content) is likely a consequence of interrupted source-sink relations leading to reduced influx of sugars into berries [45]. Although previous studies have shown transcriptomic and metabolomics changes in ripening-related processes due to viral infection [49], [50], detailed investigations on the physiological, molecular and biochemical mechanisms on how virus infection regulates berry ripening-related processes require further studies. Many of the effects on wine chemistry observed in this study, in turn, have both direct chemical and sensory implications. Further studies on cultivar-dependent responses to GLD infection across successive growing seasons and in distinct geo-climatic locations would help to elucidate the complex responses of grapevines to viral infection and discriminate host-virus interactions from that of confounding factors in the field due to climate-related variables.

## Supporting Information

S1 Fig Cumulative growing degree days (GDD) of the Roza weather station (46.3°N latitude, 119.7°W longitude) of the Washington Agricultural Weather Network (AgWeatherNet; http://weather.wsu.edu/awn.php), located close to the commercial vineyard block (46.2°N latitude, 119.8°W longitude). Approximate date of *véraison* is denoted by the grey diamond. Data was retrieved on February 25, 2014.
(TIF)
