# Supplementary figures and images for: Impacts of Grapevine Leafroll Disease on Fruit Yield and Grape and Wine Chemistry in a Wine Grape (Vitis vinifera L.) Cultivar
Source: PLoS One. 2016 Feb 26;11(2):e0149666. doi: 10.1371/journal.pone.0149666 (PMC4769264; doi:10.1371/journal.pone.0149666)

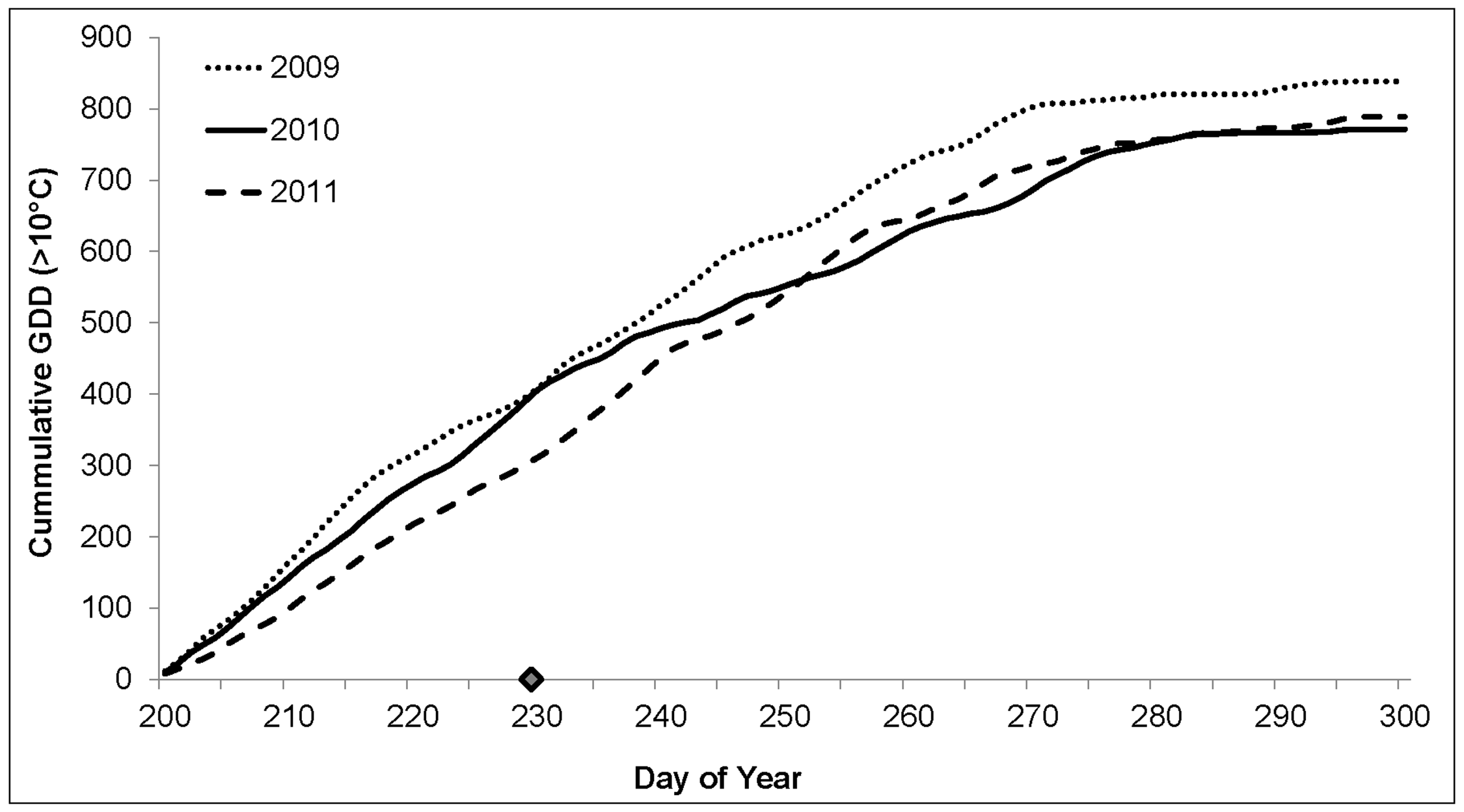

Supplement: S1 Fig — Approximate date of véraison is denoted by the grey diamond. Data was retrieved on February 25, 2014. (TIF) [file pone.0149666.s001.tif]
